# Supplementary material for: Visual impairment among diabetes patients in Ethiopia: A systematic review and meta-analysis
Source: PLoS One. 2024 May 31;19(5):e0303388. doi: 10.1371/journal.pone.0303388 (PMC11142537; doi:10.1371/journal.pone.0303388)
Supplement: S1 File — (DOCX) [file pone.0303388.s002.docx]

**Supplemental Table 2: Quality assessment of the included studies using the Joanna Briggs Institute (JBI) quality appraisal criteria**

| 1. **For cross-sectional studies** | | | | | | | | | | | | | | | | | | | | | | |
| --- | --- | --- | --- | --- | --- | --- | --- | --- | --- | --- | --- | --- | --- | --- | --- | --- | --- | --- | --- | --- | --- | --- |
| **S/N** | **Author [Year]** | **Criteria** | | | | | | | | | | | | | | | | | | | **Scores** | **Overall quality** |
|  |  | Clearly defined inclusion criteria | | Describing the study settings participants | | Valid &reliable exposure measurement | | | Objective &standard criteria for measurement | | Identified confounder | | Strategies to deal with confounder | | Valid & reliable outcome measurement | | Appropriate statistical analysis | | |  | |  |
|  | Alemayehu HB et al | Y | | Y | | Y | | | Y | | N | | Y | | Y | | Y | | | 7 | | Low risk |
|  | Alemu Mersha G et al | Y | | Y | | Y | | | Y | | N | | Y | | Y | | Y | | | 7 | | Low risk |
|  | Alemu S et al | Y | | Y | | Y | | | Y | | N | | Y | | Y | | Y | | | 7 | | Low risk |
|  | Asemu MT et al | N | | Y | | Y | | | Y | | N | | Y | | N | | Y | | | 5 | | Low risk |
|  | Chisha Y et al | N | | Y | | Y | | | Y | | N | | Y | | Y | | Y | | | 7 | | Low risk |
|  | Demilew KZ et al | Y | | Y | | Y | | | Y | | Y | | Y | | Y | | N | | | 7 | | Low risk |
|  | Ejeta A et al | Y | | Y | | Y | | | N | | N | | Y | | N | | Y | | | 5 | | Low risk |
|  | Ejigu T et al | Y | | Y | | Y | | | Y | | N | | Y | | Y | | Y | | | 7 | | Low risk |
|  | Gizaw M et al | Y | | Y | | Y | | | N | | Y | | Y | | Y | | Y | | | 7 | | Low risk |
|  | Gudina EK et al | Y | | Y | | Y | | | Y | | N | | Y | | Y | | Y | | | 7 | | Low risk |
|  | Kabtu E et al | Y | | Y | | Y | | | N | | N | | Y | | Y | | Y | | | 6 | | Low risk |
|  | Lebeta R et al | Y | | Y | | Y | | | Y | | N | | Y | | Y | | Y | | | 7 | | Low risk |
|  | Sahiledengle B et al | Y | | Y | | Y | | | Y | | N | | Y | | Y | | Y | | | 7 | | Low risk |
|  | Seid MA et al | Y | | Y | | Y | | | Y | | N | | Y | | Y | | Y | | | 7 | | Low risk |
|  | Seid MA et al | Y | | Y | | Y | | | Y | | N | | Y | | Y | | Y | | | 7 | | Low risk |
|  | Shibru T et al | N | | Y | | Y | | | Y | | N | | Y | | Y | | Y | | | 6 | | Low risk |
|  | Tesfaye DJ et al | Y | | Y | | Y | | | Y | | N | | Y | | Y | | Y | | | 7 | | Low risk |
|  | Tilahun AN et al | Y | | Y | | Y | | | Y | | N | | Y | | Y | | Y | | | 7 | | Low risk |
|  | Tilahun M et al | Y | | Y | | Y | | | Y | | N | | Y | | Y | | Y | | | 7 | | Low risk |
|  | Tilahun M et al | Y | | Y | | Y | | | Y | | N | | Y | | Y | | Y | | | 7 | | Low risk |
|  | Tsegaw A et al | Y | | Y | | Y | | | Y | | N | | Y | | Y | | Y | | | 7 | | Low risk |
|  | Worku D et al | Y | | Y | | Y | | | Y | | N | | Y | | Y | | Y | | | 7 | | Low risk |
|  | Woyessa DN et al | Y | | Y | | Y | | | Y | | N | | Y | | Y | | Y | | | 7 | | Low risk |
|  | YimamAhmed M et al | N | | Y | | Y | | | Y | | N | | Y | | Y | | Y | | | 6 | | Low risk |
|  | Zegeye AF et al | Y | | Y | | Y | | | Y | | N | | Y | | Y | | Y | | | 7 | | Low risk |
|  | Zewdu K et al | Y | | Y | | Y | | | Y | | N | | Y | | Y | | Y | | | 7 | | Low risk |
| 1. **For cohort and case-control studies** | | | | | | | | | | | | | | | | | | | | | | |
| *S/N* | ***Authors*** | | Q1 | | Q2 | | Q3 | Q4 | | Q5 | | *Q6* | | *Q7* | | *Q8* | | *Q9* | *Q10* | | | *% of* ***ʺYesʺ*** |
|  | Azeze TK et al | | N | | N | | Y | Y | | Y | | Y | | Y | | Y | | Y | Y | | | *8/10=80%* |
|  | Debele GR et al | | N | | N | | Y | Y | | Y | | Y | | Y | | N | | Y | Y | | | *7/10=70%* |
|  | ***Garoma D et al*** | | Y | | Y | | N | Y | | Y | | Y | | Y | | Y | | Y | N | | | *8/10=80%* |
|  | Gelcho GN et al | | N | | N | | N | Y | | Y | | Y | | Y | | Y | | Y | Y | | | *7/10=70%* |
|  | Kebede SA et al | | N | | N | | Y | Y | | Y | | Y | | Y | | Y | | Y | Y | | | *8/10=80%* |
|  | ***Seid K et al*** | | Y | | Y | | N | Y | | Y | | Y | | N | | Y | | Y | Y | | | *8/10=80%* |
|  | Takele MB et al | | N | | N | | Y | Y | | Y | | Y | | Y | | Y | | Y | Y | | | *8/10=80%* |
|  | Wolde HF et al | | N | | N | | Y | Y | | Y | | Y | | Y | | Y | | Y | Y | | | *8/10=80%* |

*Note: Q1,* Two groups are similar and recruited from the same population*; Q2,* Similar measurement of exposure both for exposed and unexposed groups*; Q3,* Valid and reliable measurement of exposure; Q4, Identifying confounders; Q5, Strategies to deal with confounders; Q6, Groups are free of the outcomes at the beginning; Q7, Valid and reliable measurement of outcomes; Q8, Long enough follow-up time for the occurrence of outcomes; Q9, Complete follow-up time; Q10, Strategies to address lost follow-up*; Y, yes; N, No*

**Supplemental Table 3: Risk of bias assessment of the included studies**

| **S/N** | **Author [Year]** | **Criteria** | | | | | | | | | | **Scores** | **Overall risk of bias** |
| --- | --- | --- | --- | --- | --- | --- | --- | --- | --- | --- | --- | --- | --- |
|  |  | **External validity** | | | | **Internal validity** | | | | | |  |  |
|  |  | **Q1** | **Q2** | **Q3** | **Q4** | **Q5** | **Q6** | **Q7** | **Q8** | **Q9** | **Q10** |  |  |
|  | Alemayehu HB et al | Y | Y | N | Y | Y | Y | N | Y | Y | Y | 8 | Low risk |
|  | Alemu Mersha G et al | Y | Y | Y | Y | Y | N | Y | Y | N | Y | 8 | Low risk |
|  | Alemu S et al | Y | Y | N | Y | N | Y | Y | Y | Y | Y | 8 | Low risk |
|  | Asemu MT et al | Y | Y | N | Y | Y | Y | N | Y | N | Y | 7 | Low risk |
|  | Azeze TK et al | Y | Y | N | Y | Y | Y | Y | Y | N | Y | 8 | Low risk |
|  | Chisha Y et al | Y | Y | Y | Y | N | Y | N | Y | Y | Y | 8 | Low risk |
|  | Debele GR et al | Y | Y | N | Y | Y | N | Y | Y | Y | Y | 8 | Low risk |
|  | Demilew KZ et al | Y | Y | N | Y | Y | Y | N | Y | Y | Y | 8 | Low risk |
|  | Ejeta A et al | Y | N | Y | Y | N | Y | Y | Y | Y | N | 7 | Low risk |
|  | Ejigu T et al | Y | Y | N | Y | Y | Y | N | Y | Y | Y | 8 | Low risk |
|  | Garoma D et al | Y | Y | N | Y | Y | Y | N | Y | Y | Y | 8 | Low risk |
|  | Gelcho GN et al | Y | Y | N | Y | Y | Y | N | Y | Y | N | 7 | Low risk |
|  | Gizaw M et al | N | Y | Y | Y | Y | N | Y | Y | Y | Y | 8 | Low risk |
|  | Gudina EK et al | Y | Y | N | Y | Y | Y | N | Y | Y | Y | 8 | Low risk |
|  | Kabtu E et al | Y | Y | N | Y | Y | Y | Y | N | Y | Y | 8 | Low risk |
|  | Kebede SA et al | Y | Y | N | Y | Y | Y | N | Y | Y | Y | 8 | Low risk |
|  | Lebeta R et al | Y | Y | N | Y | N | Y | Y | Y | Y | Y | 8 | Low risk |
|  | Sahiledengle B et al | Y | Y | Y | Y | Y | N | Y | Y | N | Y | 8 | Low risk |
|  | Seid K et al | Y | Y | Y | N | Y | N | N | Y | Y | Y | 7 | Low risk |
|  | Seid MA et al | Y | Y | N | Y | Y | Y | N | Y | Y | Y | 8 | Low risk |
|  | Seid MA et al | Y | Y | N | Y | Y | N | Y | Y | Y | Y | 8 | Low risk |
|  | Shibru T et al | Y | Y | N | Y | Y | Y | N | Y | Y | Y | 8 | Low risk |
|  | Takele MB et al | Y | Y | N | Y | Y | Y | N | Y | Y | Y | 8 | Low risk |
|  | Tesfaye DJ et al | Y | Y | Y | N | Y | N | Y | Y | Y | Y | 8 | Low risk |
|  | Tilahun AN et al | Y | Y | N | Y | Y | Y | N | Y | Y | Y | 8 | Low risk |
|  | Tilahun M et al | Y | Y | N | Y | Y | Y | N | Y | Y | Y | 8 | Low risk |
|  | Tilahun M et al | Y | Y | N | Y | Y | Y | N | Y | Y | Y | 8 | Low risk |
|  | Tsegaw A et al | Y | Y | N | Y | Y | N | Y | Y | Y | Y | 8 | Low risk |
|  | Wolde HF et al | Y | Y | Y | N | Y | Y | N | Y | Y | Y | 8 | Low risk |
|  | Worku D et al | Y | Y | N | Y | Y | Y | N | Y | Y | Y | 8 | Low risk |
|  | Woyessa DN et al | Y | Y | N | Y | Y | Y | N | Y | Y | Y | 8 | Low risk |
|  | YimamAhmed M et al | Y | Y | N | Y | Y | N | Y | Y | N | Y | 7 | Low risk |
|  | Zegeye AF et al | N | Y | Y | Y | Y | Y | N | Y | Y | Y | 8 | Low risk |
|  | Zewdu K et al | Y | Y | N | Y | Y | Y | N | Y | Y | Y | 8 | Low risk |

Note: Y, Yes; N, No; Q1, Representatives of the target population; Q2, Representativeness of the sampling frame; Q3, Random sampling or census; Q4, Minimal response bias; Q5, Data were collected directly; Q6, Acceptable case definition used in the study; Q7, Valid and reliable measurement; Q8, The same mode of data collection for all study subject; Q9, Appropriate length of prevalence period for parameter of interest and Q10, Appropriate numerators and denominators of interest.
