# Supplementary material for: Visual impairment among diabetes patients in Ethiopia: A systematic review and meta-analysis
Source: PLoS One. 2024 May 31;19(5):e0303388. doi: 10.1371/journal.pone.0303388 (PMC11142537; doi:10.1371/journal.pone.0303388)
Supplement: Attachment — Submitted filename: Comments to Author 1.docx [file pone.0303388.s003.docx]

Comments to Author (PONE-D-23-23377)

Thank you, I am happy for the chance to review this kind of manuscript. Here below are my comments on the manuscript.

This is a well-written and important study highlighting Visual impairment and its associated factors among diabetes patients in Ethiopia. There are aspects I have great concern-on topic synthesis

1. In almost all of your individual studies included in your meta-analysis, the topic was “prevalence and associated factors of diabetic retinopathy, but your title said “Visual impairment and its associated factors among diabetes patients in Ethiopia: systematic review and Meta-analysis”. Do you believe that the terms "visual impairment" and "diabetic retinopathy" are similar? I have noticed also an existing systematic review in Ethiopia," Diabetic retinopathy in Ethiopia: A systematic review and meta-analysis. It seems your results are overlapping, including, in part, same studies.”<https://www.sciencedirect.com/science/article/abs/pii/S1871402119302036?via%3Dihub>.

In addition, when we see your discussion, the literatures used was focuses on diabetic retinopathy rather than on visual impairment and associated factors. Are you going to compare unrelated studies?

2. Before resubmission, a person proficient in written English edits the manuscript. It is important that the message being conveyed in the manuscript is as unambiguous as possible. For instance, here are some examples of language related problems.

Abstract

Page-2 -rewrite the vague sentence “Although numerous attempts have been made to ascertain the occurrence of visual impairment and its associated factors among diabetes patients in Ethiopia, several original studies have reported incongruous results” to

Page-3-Change literatures to literature

Page-3 change ‘from’ to on

Page 3-4 change is to are (patients is needed).

P 3 Line 66-67 please rewrite or remove these sentence in your abstract conclusion i.e. “Therefore, diabetic patients should engage in regular physical activity, control their glycemic level with proper use of medications, manage co morbid hypertension, and have regular eye exams”.

3. Page 4 Line 85 to 86, I believe there are more recent sources, for instance, <https://www.researchgate.net/publication/372489279_The_burden_of_visual_impairment_among_Ethiopian_adult_population_Systematic_review_and_meta-analysis>
4. Why you limited the numbers of keywords for your search? Especially related to individuals studies included in your references like “retinopathy, diabetic retinopathy and cataract etc.”
